# A harm-threshold model for ethical organ allocation

**DOI:** 10.3389/frhs.2025.1726252

**Published:** 2025-12-01

**Authors:** Yan Jun Lin

**Affiliations:** Cornell University, College of Arts and Sciences, Ithaca, NY, United States

**Keywords:** organ allocation, medical ethics, decision theory, health policy, transplantation, MELD, utilitarianism

## Abstract

**Background:**

Allocation of scarce donor organs must balance improving overall outcomes with protecting patients at greatest near-term risk. Urgency-focused systems such as MELD (Model for End-Stage Liver Disease) are efficient in saving lives but can reduce total post-transplant survival, whereas unconstrained utility maximization risks bypassing the sickest patients.

**Methods:**

We propose Harm-Threshold Utilitarianism (HTU), which maximizes expected post-transplant benefit subject to two guardrails: (i) an epistemic threshold requiring sufficient confidence before acting on predicted differences, and (ii) a catastrophic harm threshold that blocks bypassing candidates at high short-term waitlist mortality risk for only marginal or uncertain gains. Using de-identified U.S. liver transplant registry data, we performed proof-of-concept simulations of offer-like pools, comparing HTU with MELD-based selection. Outcomes included Kaplan–Meier curves and 5-year restricted mean survival time (RMST). Sensitivity analyses varied the harm threshold and confidence level.

**Results:**

HTU transparently reorders candidates relative to MELD while preserving alignment on broad priorities. In simulated pools, HTU-selected recipients achieved higher post-transplant survival; mean RMST improved by approximately 0.25 years per transplant (about three months) at baseline settings. Varying the catastrophic harm threshold produced a clear urgency–efficiency frontier: tighter thresholds selected more urgent patients with smaller gains, while looser thresholds increased gains but allowed more bypasses of urgent candidates.

**Conclusions:**

HTU operationalizes a tunable, ethically explicit trade-off between benefit and protection of the worst-off. By encoding precaution (confidence threshold) and a non-negotiable floor against catastrophic harm, HTU offers measurable efficiency gains without sacrificing fairness, and reframes policy choices as transparent parameters open to review.

## 1 Introduction

Organ transplantation presents a central ethical dilemma: when donor organs are scarce, by what principle should recipients be chosen? Current systems of allocation, such as the Model for End-Stage Liver Disease (MELD) and the Lung Allocation Score (LAS), prioritize urgency with limited adjustment for post-transplant outcomes (1). These urgency-based rules have saved many lives by ensuring access for patients at highest immediate risk. However, prioritizing those at the brink of death can reduce overall life-years gained, as such patients often have limited survival after transplantation. Utilitarian proposals push in the opposite direction: maximize life-years or QALYs by favoring candidates with better prognoses (2). Simulations show that this approach could yield more overall survival (3), but the moral cost is steep—such policies routinely pass over the sickest patients, effectively denying them any chance (4). The public is unlikely to accept a system that appears to sacrifice the worst-off for incremental gains. What remains are hybrid rules, like MELD with urgency–benefit weights or waiting-time tiebreakers. These compromises vary across organs, are not clearly justified, and risk losing legitimacy by lacking a principled ethical basis (1).

We introduce Harm-Threshold Utilitarianism (HTU) as a framework that makes the trade-offs explicit. HTU retains the utilitarian goal of maximizing expected post-transplant survival, but only within two boundaries. An epistemic threshold prevents acting on weak or uncertain predictions. A catastrophic harm threshold prohibits allocations that would leave a patient at imminent risk of death untreated for the sake of a marginal gain in another. These guardrails incorporate basic deontological commitments—precaution and protection of the worst-off—into a utilitarian process. This ethical foundation is not incidental; it responds directly to longstanding critiques of utilitarian reasoning in medicine. Classic discussions of act utilitarianism underscore its willingness to sacrifice individuals for aggregate welfare, epitomized by the “transplant surgeon” thought experiment in which one healthy person is killed to save five others (5). Such reasoning highlights the danger of reducing persons to instruments of collective benefit. Smart has argued that utilitarianism, when applied without constraint, can demand choices that erode personal integrity and the stability of moral rules (6). Nozick takes this further, contending that any framework that permits treating individuals as mere means fails to respect the rights and inviolability that persons inherently possess (7). Rawls similarly defends the idea that each person holds a moral status rooted in justice that cannot be overridden even by large social gains (8). Extending this line of thought to health care, Daniels maintains that fairness requires protecting the worst-off to preserve equality of opportunity (9).

Harm-Threshold Utilitarianism incorporates these insights into a practical decision rule. By requiring robust evidence before privileging one candidate over another, it guards against decisions based on weak or speculative predictions. By prohibiting allocations that would deny treatment to patients facing catastrophic near-term risk for only marginal or uncertain benefit elsewhere, it ensures that efficiency is never pursued at the cost of fairness or dignity. In this way, HTU does not reject utility, but channels it within ethical boundaries that preserve both individual rights and public trust. By reframing allocation as a constrained optimization, HTU turns implicit value judgments into explicit thresholds. This makes trade-offs auditable, adaptable across organs, and open to public scrutiny. Our aim in this paper is to formalize HTU, demonstrate its logic using retrospective transplant data, and show how it can achieve measurable survival gains without sacrificing fairness.

### 1.1 Utilitarian aims vs. ethical constraints

Classical utilitarianism, as developed by Bentham and Mill, holds that actions and policies should be judged by their consequences for overall well-being. In transplantation, this translates into a straightforward rule: organs should be given to those who are expected to gain the greatest total benefit, whether measured in survival time or quality-adjusted life years (QALYs). The principle of utility is explicitly recognized in organ allocation ethics, where maximizing total lives or life-years saved is treated as a central objective (10).

Yet unconstrained utilitarianism has long been criticized for colliding with other core principles, including justice, fairness, and respect for persons (11). Pure act utilitarianism, which evaluates each decision by its immediate net benefit, can endorse conclusions that appear morally repugnant in practice. The classic illustration is the “transplant surgeon” thought experiment: killing one healthy individual to save five others through organ harvest would maximize utility, but violates fundamental moral intuitions about rights and dignity (5).

Philosophers have responded with refinements. Rule utilitarianism proposes that general rules—such as “do not kill innocents” or “give priority to the worst-off”—are justified because they maximize welfare in the long run while maintaining consistency and moral integrity (6). In organ allocation, urgency-based rules like “sickest-first” can be seen as such principles: they sacrifice some efficiency in individual cases, but preserve fairness and predictability across the system.

Threshold deontology introduces a further nuance: deontological constraints (such as prohibitions against sacrificing an individual) should not be overridden except in cases where the consequences of adhering to them would be catastrophically severe. This maps directly onto transplant ethics. Denying a dying patient a transplant in order to achieve only a modest gain in another patient’s expected survival seems unjustifiable. But if the alternative benefit were vastly greater and highly certain, some might argue that strict urgency rules could be permissibly set aside.

Harm-Threshold Utilitarianism builds on this philosophical lineage by embedding such limits directly into the allocation rule. It formalizes the intuition that utilitarian gains can matter, but only when they clear explicit thresholds of confidence and proportionality. In doing so, HTU translates abstract ethical debate into operational criteria that can guide real-world allocation policies.

## 2 Harm-threshold utilitarianism: formal definition

Formally, assume each transplant candidate i has an estimated utility $U_{i}$ (expected quality-adjusted life years gained from the transplant). The index i ranges over all eligible patients in the decision set. A classical utilitarian policy would simply allocate the organ to the candidate k with the highest $U_{i}$:

$$k=\arg\max_{i}U_{i},$$

where arg⁡max means “the index i that produces the largest value of $U_{i}$.” This yields the candidate’s identity, not the utility value itself.

**Figure 1:**
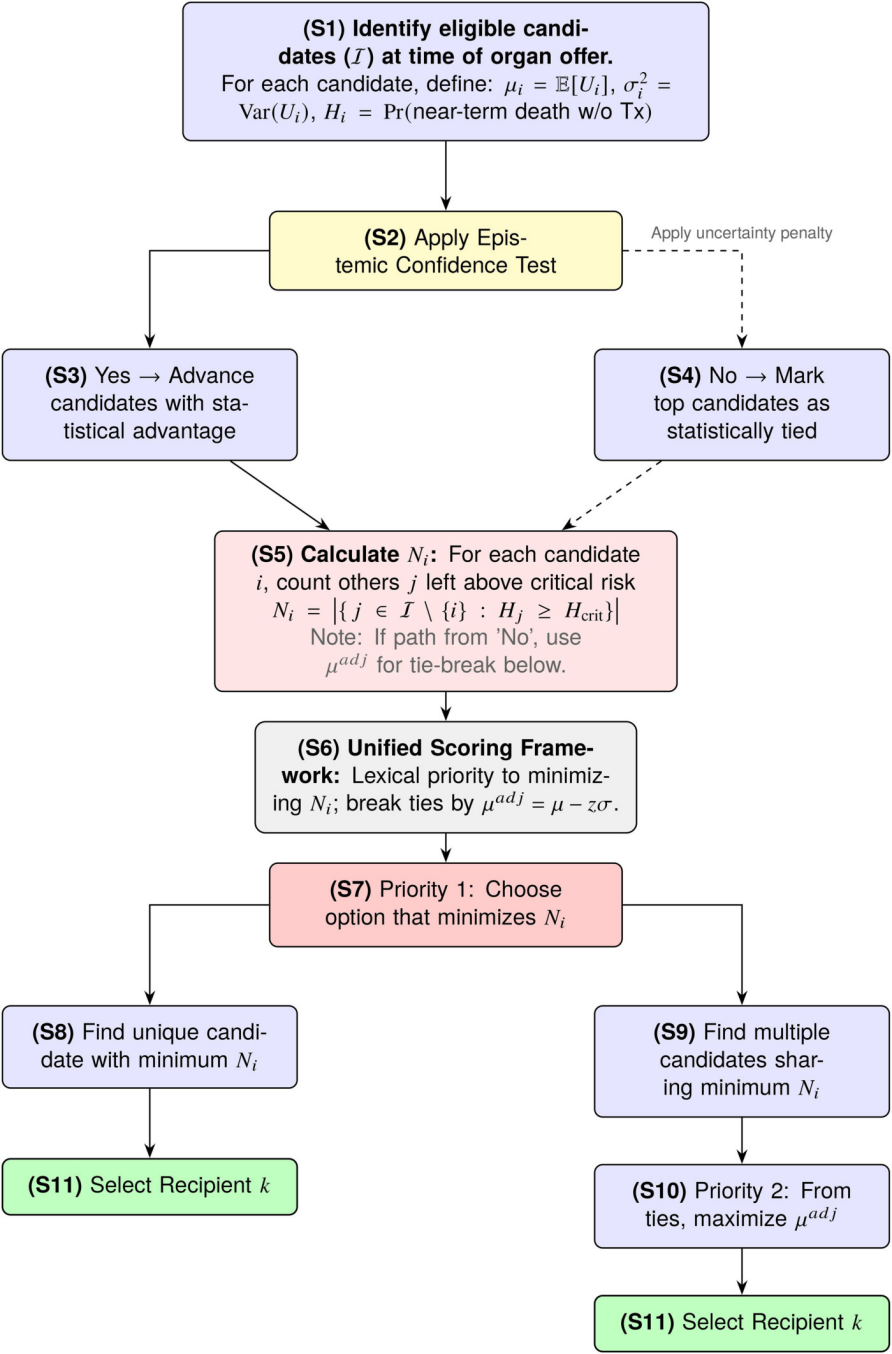
The HTU decision flow. An Epistemic Screen first filters for candidates with a robust utility advantage. A Harm Threshold then prioritizes minimizing catastrophic harm ($N_{i}$), using uncertainty-adjusted benefit ($\mu^{adj}$) as the tie-breaker.

HTU modifies this decision by introducing two sequential constraints that filter or adjust the choice, as illustrated in the decision flowchart in Figure 1:

Here $U_{i}$ is treated as a random variable (**S1**) to account for the fact that our prediction of benefit is uncertain. Each $U_{i}$ has an associated mean $\mu_{i}$ (the point estimate of benefit) and variance $\sigma_{i}^{2}$ (the predictive uncertainty around that estimate). This variance term plays a critical role: even if two candidates have similar $\mu_{i}$, a larger $\sigma_{i}$ will reduce our confidence in that candidate being truly better. HTU requires that a candidate’s apparent advantage be statistically robust (**S2**). In practice, candidate i is chosen over candidate j only if the probability that $U_{i}$ exceeds $U_{j}$ is greater than 1−α (**S3**):

$$P(U_{i}>U_{j})>1-\alpha ,$$

where α is a pre-specified tolerance for error (e.g., α=0.05 for a 95% confidence requirement). For normally distributed uncertainties, this condition becomes:

$$P(U_{i}>U_{j})=\Phi \left(\frac{\mu_{i}-\mu_{j}}{\sqrt{\sigma_{i}^{2}+\sigma_{j}^{2}}}\right)>1-\alpha ,$$

where Φ(⋅) is the standard normal cumulative distribution function. The numerator $\left(\mu_{i}-\mu_{j}\right)$ represents the observed gap in mean benefit, while the denominator $\sqrt{\sigma_{i}^{2}+\sigma_{j}^{2}}$ reflects total uncertainty in that gap. This ratio is essentially a “signal-to-noise” measure: larger gaps and smaller uncertainty both increase the probability. If no candidate’s advantage meets this confidence threshold against all others, the set of top candidates is considered tied (**S4**), and the decision process then defers to Step 2 to break the tie.

Let $H_{i}$ denote the predicted probability that patient i will die in the short-term without receiving an organ. This probability can be estimated from historical waitlist data, clinical risk models, or other prognostic tools.

We set a critical threshold $H_{\mathrm{crit}}$ (e.g., 0.30). Any patient with $H_{i}\geq H_{\mathrm{crit}}$ is considered to be in catastrophic danger. A lower $H_{\mathrm{crit}}$ classifies more patients as catastrophically ill, while a higher value classifies fewer. For each possible recipient i, we calculate (**S5**):

$$N_{i}=\left|\left\{\;j\in I\setminus \{i\}\;:\;H_{j}\geq H_{\mathrm{crit}}\right\}\right|,$$

where I is the set of all eligible patients and |⋅| denotes the number of elements in the set (cardinality). In practice, $N_{i}$ counts the number of other patients who would remain above the critical risk threshold if patient i were selected to receive the organ. Because the patient pool and their associated $H_{j}$ values can change over time, $N_{i}$ is inherently dynamic and must be recalculated for each allocation decision. The selection rule is (**S6**):

$$k=\arg\min_{i}\left(N_{i},\;-\mu_{i}^{adj}\right),$$

where arg⁡min means “choose the index i with the smallest value”— first minimize $N_{i}$ (**S7**), which may identify a unique candidate (**S8**) or multiple candidates (**S9**); then among ties, maximize the uncertainty-adjusted expected benefit $\mu_{i}^{adj}$ (**S10**) to make the final selection (**S11**). Here $\mu_{i}^{adj}=\mu_{i}-z\sigma_{i}$ with $z=\Phi^{-1}(1-\alpha )$.

## 3 Methods

### 3.1 Framework and study population

Our objective was to evaluate Harm-Threshold Utilitarianism (HTU) as a decision framework rather than to develop a definitive predictive model for transplantation. The central aim was to show that explicit thresholds for epistemic confidence and catastrophic harm can structure allocation in a way that is systematic, fair, and adaptable to different ethical priorities. To test this, we applied HTU to registry-based simulations, constructing candidate pools that isolate the allocation logic and allow direct comparison with MELD. This design demonstrates how HTU reorders candidates and clarifies the trade-offs it enforces. Crucially, the framework not only makes value judgments explicit and auditable, but also shows that respecting these ethical constraints does not come at the cost of efficiency: even in retrospective data, HTU yields modest gains in predicted survival while advancing transparency and normative clarity.

To that end, we used de-identified registry data from the OPTN STAR files (2002–2025) under a Data Use Agreement with OPTN/UNOS. Data were accessed on August 22, 2025; there was no access to identifiable information at any time. The cohort included adult liver transplant candidates listed under the MELD-based allocation system, with linked demographic, laboratory, and outcome data. Each candidate was assigned an estimated post-transplant survival (mean μ) with associated predictive uncertainty (σ), as well as a short-term mortality risk without transplant (H). These quantities operationalize the three elements central to HTU: expected benefit, uncertainty, and near-term harm. All analyses were conducted in MATLAB (R2024a, MathWorks).

### 3.2 Outcome prediction, calibration, and allocation metrics

Post-transplant survival predictions formed the basis for utility ranking. Candidate-level features and outcomes were drawn from linked OPTN STAR files (LIVER_DATA, WLHISTORY, FOLLOWUP, DONOR) and merged with precomputed estimates of expected post-transplant survival in years (μ) and associated prediction uncertainty (σ). Model performance was assessed by stratifying patients into μ-deciles and plotting Kaplan–Meier survival curves to verify calibration. To evaluate how HTU compares with the existing MELD framework, we expressed both systems as rank orderings of candidates. Concordance between the two was quantified with Kendall’s τ and Spearman’s ρ, while scatterplots were generated to visualize overall rank agreement. We further examined the distribution of rank shifts, defined as HTU minus MELD rank. These analyses characterized the extent to which HTU reorders candidates relative to MELD. Near-term mortality without transplant, $H_{i}$, is operationalized as predicted 90-day waitlist mortality using time-updated covariates available at the offer time. The baseline catastrophic harm threshold is $H_{\text{crit}}=0.30$, chosen to approximate the upper-quartile of short-term risk in this cohort; sensitivity analyses sweep $H_{\text{crit}}\in \{0.20,0.30,0.40\}$ and beyond to characterize urgency–efficiency trade-offs.

### 3.3 Sensitivity to policy parameters

HTU was operationalized as a sequential, uncertainty-aware rule. For each offer, an epistemic screen was first applied to avoid acting on low-confidence differences in expected post-transplant survival: with $z=\Phi^{-1}(1-\alpha )$, we defined $\mu^{adj}=\mu -z\sigma$ and treated candidates as tied unless pairwise gaps exceeded this uncertainty margin. Within the screened set, a harm safeguard prioritized candidates whose near-term waitlist risk exceeded a fixed threshold ($H\geq H_{\text{crit}}$) to prevent bypass of high-risk patients. Residual ties after applying the harm threshold were resolved by selecting the candidate with the largest $\mu^{adj}$. For cohort-level rankings, we used a scalable surrogate consistent with this logic, sorting lexicographically by a harm proxy (fewest others remaining above $H_{\text{crit}}$) and then by $\mu^{adj}$. Baseline parameters were set to α=0.05 and $H_{\text{crit}}=0.30$.

To examine the influence of these policy knobs, we conducted sensitivity analyses by varying $H_{\text{crit}}$ from 0.10 to 0.70 while holding α fixed, and by adjusting α around its baseline value. For each setting, we recorded both the mean survival benefit of the HTU-selected recipients and the proportion of urgent candidates chosen, enabling construction of urgency–efficiency trade-off curves.

### 3.4 Simulated allocation outcomes

We evaluated the practical impact of HTU vs. MELD using simulated “offer-like” pools. From the study cohort we generated 2,000 independent pools of 10 compatible candidates each, sampled with replacement. Within each pool, the MELD policy selected the candidate with the highest MELD score, while HTU applied its sequential rule (epistemic screen, harm safeguard, and $\mu^{adj}$ tie-break) under baseline settings of α=0.05 and $H_{\text{crit}}=0.30$. To isolate the effect of ranking criteria, simulations enforced ABO and basic donor–recipient compatibility but did not model regional logistics or MELD exception pathways. For each policy and parameter setting, we tracked the characteristics of the selected recipients and estimated their post-transplant outcomes. Predicted outcomes were summarized with Kaplan–Meier survival curves and restricted mean survival time (RMST) up to five years. By comparing RMST across policies, we quantified the expected gain in post-transplant survival attributable to HTU relative to MELD.

## 4 Results

### 4.1 Predicted utility model validation

**Figure 2:**
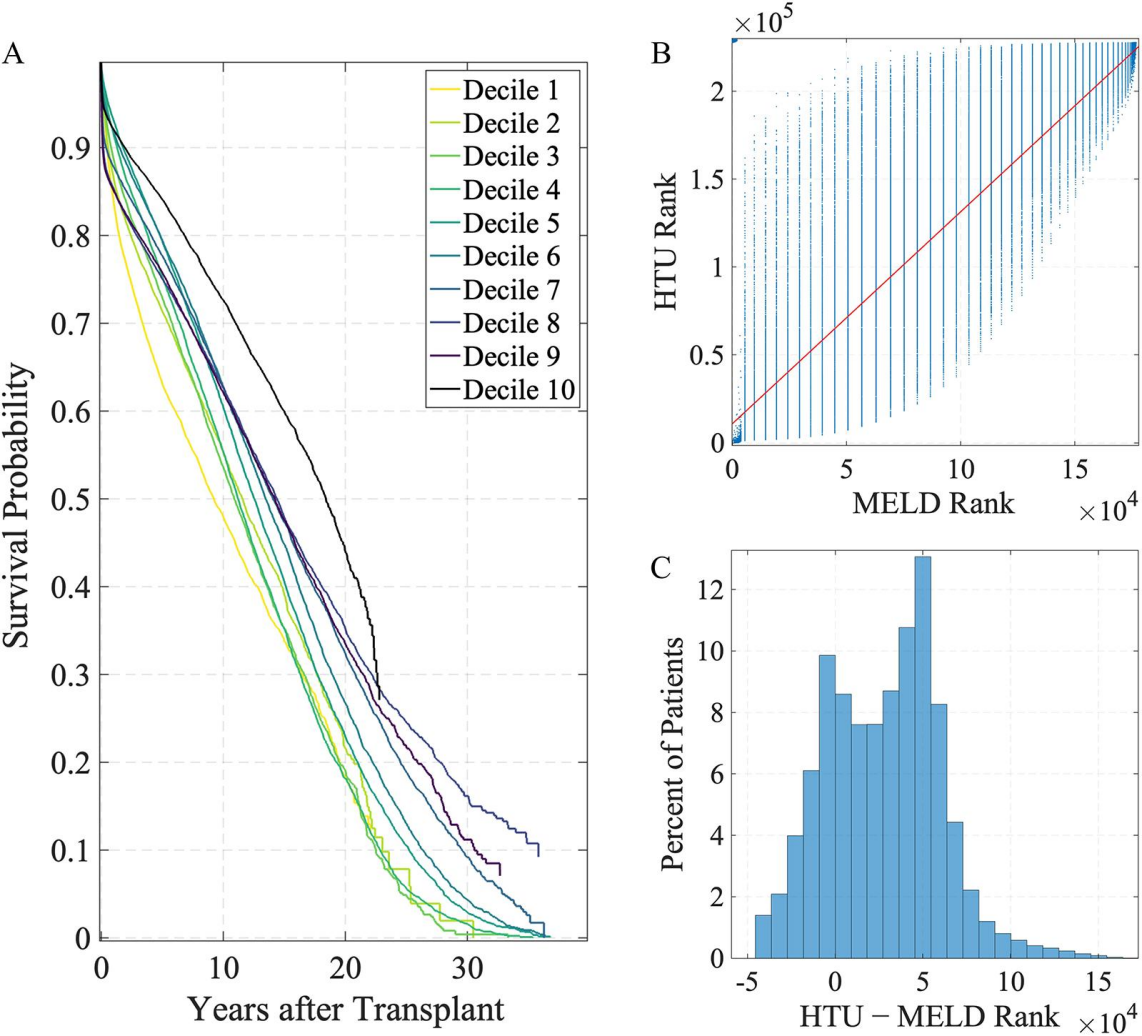
Survival stratification and priority ranking under HTU and MELD. **(A)** Kaplan–Meier survival curves stratified by deciles of predicted 5-year survival (μ) using the HTU framework, where decile 1 represents the lowest predicted survival and decile 10 the highest. **(B)** MELD vs. HTU priority ranking. The red line shows the least-squares fit; lower rank values (closer to 1) correspond to higher priority for transplant. Kendall’s τ=0.720, Spearman’s ρ=0.857 (n=1,76,823). **(C)** Distribution of rank shifts (HTU rank−MELD rank); negative values indicate higher priority under HTU.

The HTU allocation framework relies on a predictive model of post-transplant survival, which we evaluated on the historical data. Kaplan–Meier survival curves stratified by deciles of predicted 5-year survival (μ) demonstrate modest separation between groups (Figure 2A). By 5 years, the highest-μ decile (Decile 10) retains approximately 85% survival, whereas the lowest-μ decile (Decile 1) is just above 60%. Adjacent lower deciles exhibit only modest gaps and some overlap. This pattern confirms that while the model is imperfect at distinguishing between patients of less urgent prognosis, it reliably separates those at the extremes: patients predicted by HTU to have the best outcomes do in fact survive substantially longer than those predicted to have the worst. Expressing both HTU and MELD priorities as ranks (1 = highest priority) reveals strong positive concordance between the two systems (Spearman’s ρ=0.857, Kendall’s τ=0.720, n=1,76,823).

The scatterplot (Figure 2B) shows a clear upward trend, indicating that in broad strokes the two systems agree on which patients look favorable. The vertical banding reflects the discreteness of MELD (integer scores from 6 to 40), which collapses large groups of patients into identical ranks. HTU, by contrast, uses continuous predictions of post-transplant survival (μ) with uncertainty and harm constraints, breaking many of these ties and spreading patients smoothly within each MELD stratum. The histogram (Figure 2C) displays the distribution of rank shifts, defined as HTU rank minus MELD rank. This distribution spans tens of thousands of positions, with negative values indicating higher HTU priority. MELD and HTU align on broad trends, but HTU reshuffles the very top, elevating patients with better predicted survival otherwise buried in MELD ties. This calibration step demonstrates that the survival predictions underlying HTU are sufficiently credible to support evaluation of policy frameworks, even if they are not intended as definitive clinical prognostic models (2, 3).

### 4.2 Sensitivity to policy parameters

**Figure 3:**
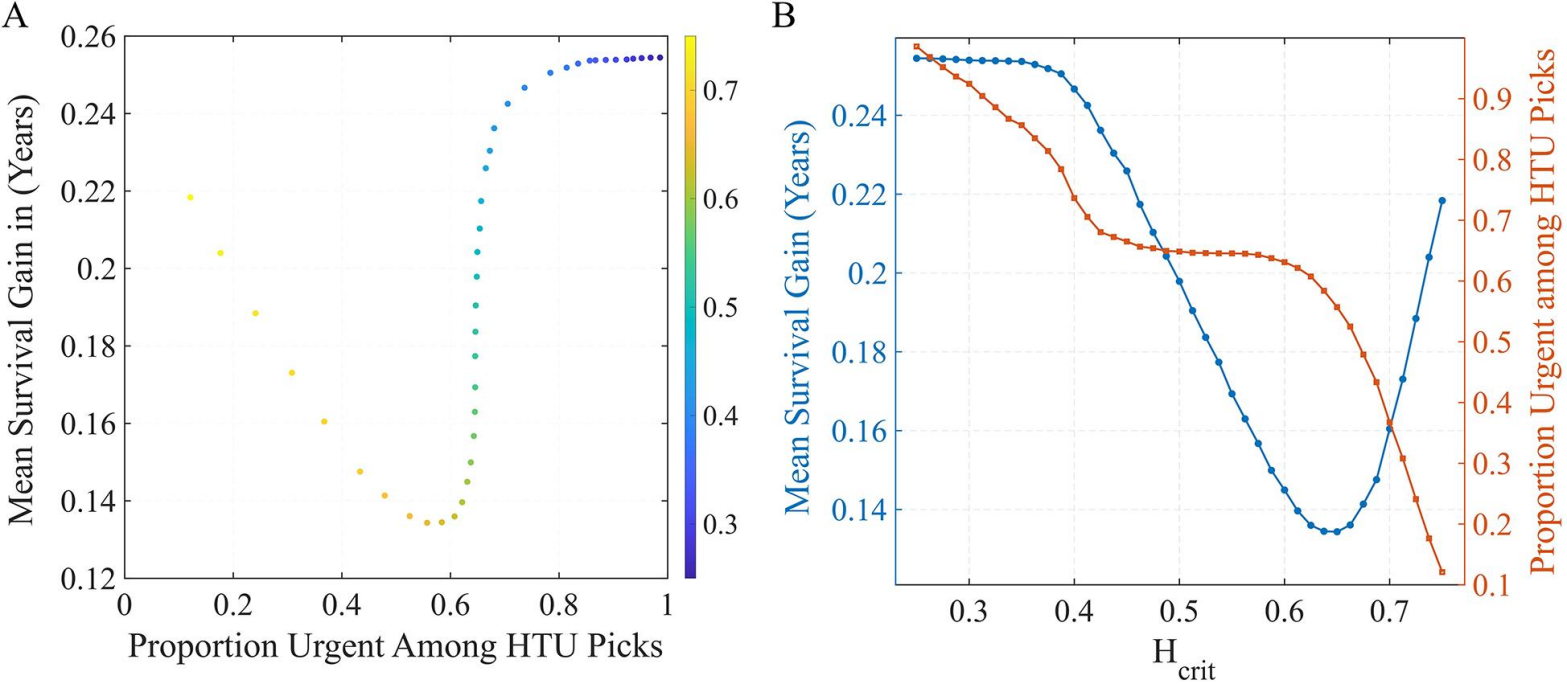
Sensitivity of HTU performance to policy settings. **(A)** Trade-off frontier between survival gain and urgency alignment across harm thresholds $H_{\text{crit}}$ (color scale). **(B)** One-way sweep of $H_{\text{crit}}$ at fixed α=0.05: blue (left axis) = mean survival gain; red (right axis) = fraction of urgent recipients chosen by HTU. Lower $H_{\text{crit}}$ preserves urgency but reduces gains, while higher $H_{\text{crit}}$ increases gains but selects fewer urgent patients.

Because HTU is designed as a tunable ethical framework, we examined how outcomes shift across different threshold values. This directly addresses longstanding concerns about transparency in balancing urgency and efficiency in allocation (1, 4). Before evaluating outcomes, we first examined how HTU performance depends on its key parameters: the harm threshold $H_{\text{crit}}$ and epistemic confidence level α. These knobs govern the trade-off between efficiency (expected life-years gained) and urgency alignment (how often HTU selects an urgent patient). Figure 3 summarizes these effects. Panel A plots the mean survival gain (in years) against the fraction of urgent recipients chosen under HTU, with points colored by the harm threshold $H_{\text{crit}}$.

As $H_{\text{crit}}$ increases, the policy is permitted to bypass more urgent patients, raising average survival gains but lowering the proportion of urgent selections. Panel B fixes α=0.05 and sweeps $H_{\text{crit}}$: the blue curve (left axis) shows expected survival gain. Tighter harm aversion (lower $H_{\text{crit}}$) preserves urgency but reduces efficiency; looser thresholds do the opposite. Across this range, the epistemic confidence parameter α plays a secondary role, fine-tuning the stringency of the uncertainty screen but with less influence than $H_{\text{crit}}$. At the baseline setting $H_{\text{crit}}=0.30$, HTU captures a substantial portion of the attainable life-year benefit (about +0.25 years on average) while still allocating to urgent candidates in many cases—representing a balanced operating point on the urgency–efficiency frontier. These findings illustrate that the harm threshold functions as a transparent policy lever: it allows decision-makers to set the acceptable balance between maximizing life-years and prioritizing the sickest patients, turning implicit value judgments into explicit, auditable parameters.

### 4.3 Simulated allocation in offer pools

To evaluate the practical consequences of these policy differences, we replayed organ offers in simulated candidate pools. We then compared the selections made under the historical MELD-based rule vs. HTU with the baseline parameters (α=0.05, $H_{\text{crit}}=0.30$).

**Figure 4:**
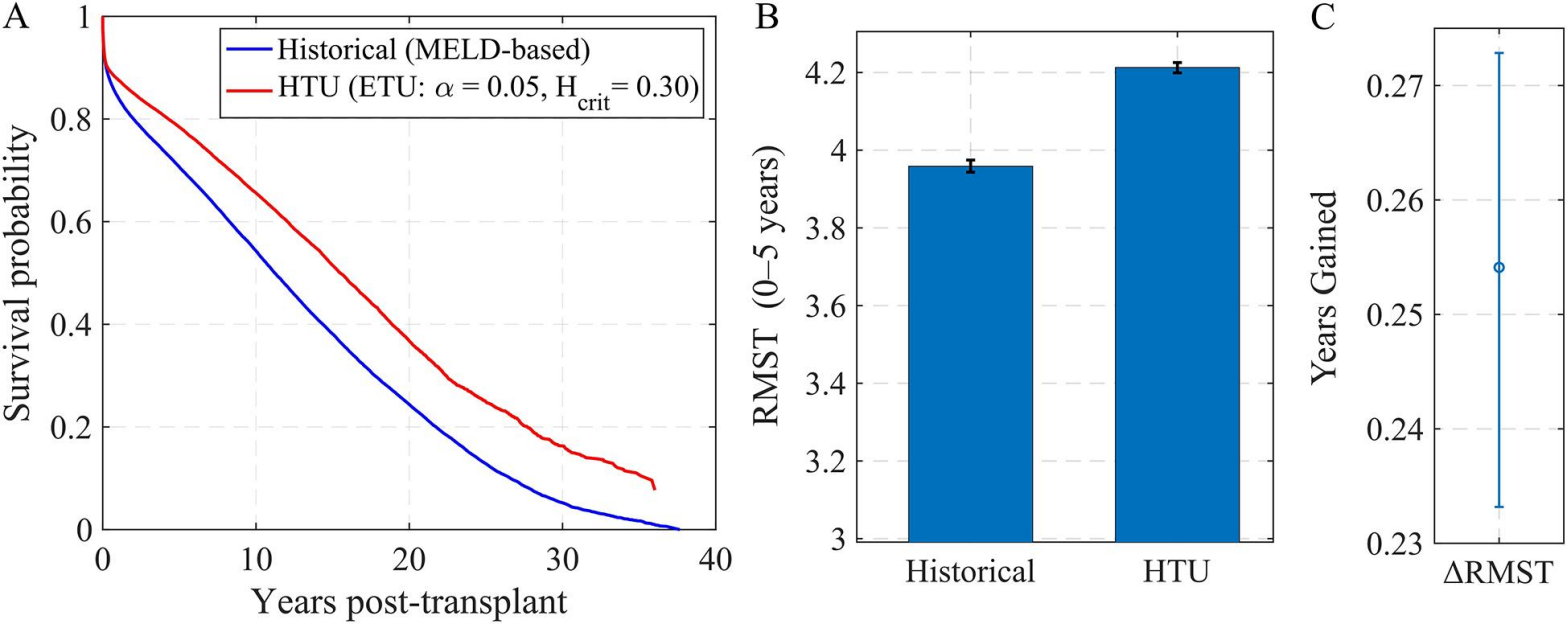
Simulated offer pools: outcomes under HTU vs. MELD-based allocation. **(A)** Kaplan–Meier post-transplant survival for recipients selected by MELD (blue) vs. HTU with α=0.05, $H_{\text{crit}}=0.30$ (red). **(B)** Restricted Mean Survival Time (0–5 years) under each policy (mean ± SE). **(C)** Difference in RMST (HTU − MELD) across simulated pools.

HTU consistently selected candidates with higher predicted post-transplant survival, leading to measurable gains in expected life-years. Figure 4A shows Kaplan–Meier curves for the recipients chosen by each policy: the HTU curve (red) lies above the MELD-based rule (blue) throughout follow-up. In Figure 4B, the restricted mean survival time (RMST) over 0–5 years is higher under HTU, and Figure 4C shows the corresponding difference. On average, HTU yields an additional ≈0.25 years (about three months) of 5-year survival per transplant compared to MELD. While this magnitude is modest, the key point is that incorporating explicit ethical thresholds does not reduce efficiency and, in fact, produces measurable improvements in outcomes.

## 5 Discussion

Harm-Threshold Utilitarianism (HTU) provides a principled alternative to the ad hoc blends of urgency and utility that dominate current allocation policies. Systems like MELD make real ethical choices but often bury those choices inside complex scoring rules that lack transparency (1, 12). HTU brings those choices to the surface. It does so by specifying, in advance, two thresholds that any decision must respect. The first is an epistemic threshold: only act on a predicted advantage when the evidence is strong enough to warrant it. The second is a harm threshold: do not pass over a patient facing clear, near-term catastrophic risk for the sake of a small or uncertain gain in someone else. In short, HTU pursues better outcomes, but only when predictions are robust and only within limits that protect those in greatest danger. This pattern reflects the basic idea behind threshold approaches in deontological ethics: moral boundaries constrain, and sometimes override, aggregate gains (13).

To see what this means in practice, consider how HTU handles common edge cases. When two candidates look similar on predicted benefit, the epistemic threshold treats them as effectively tied rather than pretending to a precision the model does not have. When one candidate is markedly better by prediction—but another is at very high short-term risk—the harm threshold prevents a bypass unless the predicted gain is both meaningful and well-supported. These are not after-the-fact justifications. They are ex ante rules that apply the same way across patients, organs, and time. In effect, HTU separates two questions that current point systems often collapse: Is the benefit difference real enough to matter? Even if it is, is it permissible to let benefit decide in the face of catastrophic near-term risk?

Our simulations are intended as a proof of concept rather than a definitive clinical model. They demonstrate that HTU can be implemented using real registry data, and that its two thresholds function as transparent policy levers that can be clearly understood and adjusted. Importantly, adding these safeguards does not reduce efficiency. The survival gains observed are modest, but they provide reassurance that fairness need not come at the expense of efficiency (4, 14). In some cases, making thresholds explicit can even improve performance by ensuring that claims of benefit are acted on only when the supporting evidence is strongest.

Transparency is one of HTU’s central strengths. In existing systems, the trade-offs that drive allocation are often buried inside lengthy formulas, and the resulting decisions are made case by case without consistent guiding principles. Small adjustments to point weights or exceptions can shift priorities in ways that are difficult to trace or justify. HTU replaces this opacity with a small set of thresholds that apply consistently across patients and cases. Because these parameters are visible, they can be explained, defended, and revised. This clarity matters for legitimacy and public trust in priority setting (9, 15, 16). It allows reviewers, clinicians, and patients to see why a candidate was chosen, and to trace the decision back to published thresholds rather than opaque computations. If those thresholds appear misguided, they can be debated and adjusted without dismantling the entire system.

HTU also addresses calls for greater stakeholder involvement in the governance of scarce medical resources (16). Unlike opaque point systems, it offers concrete decision points for deliberation: the level of confidence required before predicted benefit may guide selection, and the level of near-term risk that warrants categorical protection. These thresholds can be tailored to different organs, patient populations, or policy environments, and they can be revisited as evidence improves or as social values evolve. HTU is therefore not a static formula but a policy instrument that supports revision, accountability, and public dialogue. By making value choices explicit, it shifts debates from hidden weightings inside a formula to open discussions about the settings of key parameters. This makes allocation rules easier to justify to patients, clinicians, and the public, and easier to adjust as medicine advances.

### 5.1 Limitations

This study focused on liver transplantation, which combines high waitlist mortality with a sufficiently large sample size; application to other organs will be needed to test HTU’s generalizability. Our evaluation relied on retrospective registry data and simulated candidate pools rather than full historical replays of organ offers. This simplification allowed us to isolate allocation logic, but it omits real-world dynamics such as geographic policies, MELD exception pathways, and timing of organ availability. In addition, estimates of utility and harm remain model-dependent and may encode bias over time. Our predictive model was not designed as a definitive clinical tool, and its calibration is imperfect, particularly in mid-range cases where predictions overlap. The epistemic screen and harm threshold therefore reflect proof-of-concept rather than validated clinical cutoffs. Moreover, our analysis did not address how clinicians might respond to threshold-based rules in practice, nor how implementation could affect decision-making, trust, or donation behavior.

Equity considerations also remain preliminary. While we provided descriptive subgroup analyses, we did not optimize thresholds to address disparities by race, sex, or diagnosis, and the possibility of unequal impacts requires further study. Finally, downstream system-level effects—such as how HTU might interact with waitlist dynamics, organ acceptance practices, or evolving allocation policies—were not modeled here. Future work should extend HTU to prospective or hybrid pilots in which thresholds ($H_{\text{crit}}$, α) are set with input from clinicians, patients, and ethicists. Such work should also evaluate impacts across different demographic groups, transplant centers, and organ systems, and test how HTU functions under real-time logistical and clinical constraints.

## 6 Conclusion

Harm-Threshold Utilitarianism offers a new way of thinking about organ allocation. Rather than relying on ad hoc blends of urgency and benefit, it makes value trade-offs explicit through thresholds for epistemic confidence and catastrophic harm. These thresholds turn implicit ethical choices into clear rules that can be defended, debated, and revised. In doing so, HTU embodies a form of precautionary consequentialism: it permits utility to guide allocation only when predictions are strong and only within limits that protect those at highest near-term risk.

Ethically, HTU safeguards those at highest risk while still permitting utility to govern when predictions are robust. It integrates precaution with benefit in a way that aligns with both moral intuitions and practical needs. Practically, it is straightforward to implement on registry data, simple to monitor in operation, and adaptable across organs and eras. It can reduce the arbitrariness of current point systems by replacing case-by-case exceptions with rules that are consistent, transparent, and auditable. Its value lies not only in the modest survival gains it produces, but also in the way it writes core ethical commitments into clear, reviewable criteria that can be read, debated, and improved over time.

Our simulations show that this framework is feasible to implement with registry data and that it preserves efficiency while strengthening fairness and transparency. The survival gains observed are modest, but the deeper contribution lies in demonstrating that ethical commitments can be embedded directly into allocation rules. By reframing prioritization as a transparent, rule-based process, HTU provides policymakers with an adaptable tool that can be tuned across organs and clinical contexts. As predictive models evolve and public values shift, HTU offers a principled and flexible foundation for organ allocation. It is rigorous enough to improve outcomes, yet constrained enough to remain ethically defensible and publicly legitimate.

## Data Availability

The datasets presented in this article are not readily available because the dataset used in this study originates from the OPTN/UNOS Standard Transplant Analysis and Research (STAR) files and is subject to a Data Use Agreement that prohibits public sharing of individual-level data. Access to the raw data is restricted to approved researchers through the OPTN/UNOS application process. Only de-identified, aggregate, or simulated results may be shared by the authors upon reasonable request. Requests to access the datasets should be directed to https://optn.transplant.hrsa.gov/data/view-data-reports/request-data/.
